# Interhemispheric Acute Subdural Hematoma Secondary to Falx Meningioma: A Case Report

**DOI:** 10.7759/cureus.54886

**Published:** 2024-02-25

**Authors:** Kiyotaka Ogasawara, Yuhei Ito, Tsuyoshi Ichikawa, Kyouichi Suzuki

**Affiliations:** 1 Department of Neurosurgery, Japanese Red Cross Fukushima Hospital, Fukushima, JPN

**Keywords:** supplementary motor cortex, open craniotomy, meningioma hemorrhage, acute subdural hematoma, falx meningioma

## Abstract

This report describes an unusual case of falx meningioma associated with acute subdural hematoma, which is a rare presentation. A 76-year-old woman presented with right-sided hemiparesis and a known falx meningioma that had rapidly increased in volume over the previous year. Computed tomography revealed interhemispheric and left-hemispheric acute subdural hematomas. Preoperative embolization and surgical tumor removal were performed to improve the symptoms, and pathological examination of the tumor revealed transitional meningioma (WHO Grade I). The patient's paresis symptoms improved postoperatively. This report provides valuable insights into the management and outcomes of falx meningioma with acute subdural hematoma, suggesting aggressive surgery to improve postoperative recovery.

## Introduction

Meningiomas presenting with hemorrhage are rare, and meningiomas with subdural hematomas are even rarer [1]. Particularly, there are only eight reported cases of interhemispheric acute subdural hematoma in falx meningiomas [2-9], and its pathogenesis and optimal treatment are unclear. Here, we report a case of falx meningioma that developed an acute subdural hematoma during follow-up imaging. The patient underwent surgical removal of the tumor and had a favorable outcome.

## Case presentation

A 76-year-old woman who presented with a headache and acute progressive right-sided hemiparesis was transferred to our emergency department. There was no history of recent head trauma or consumption of oral antiplatelet or anticoagulant medication.

**Figure 1:**
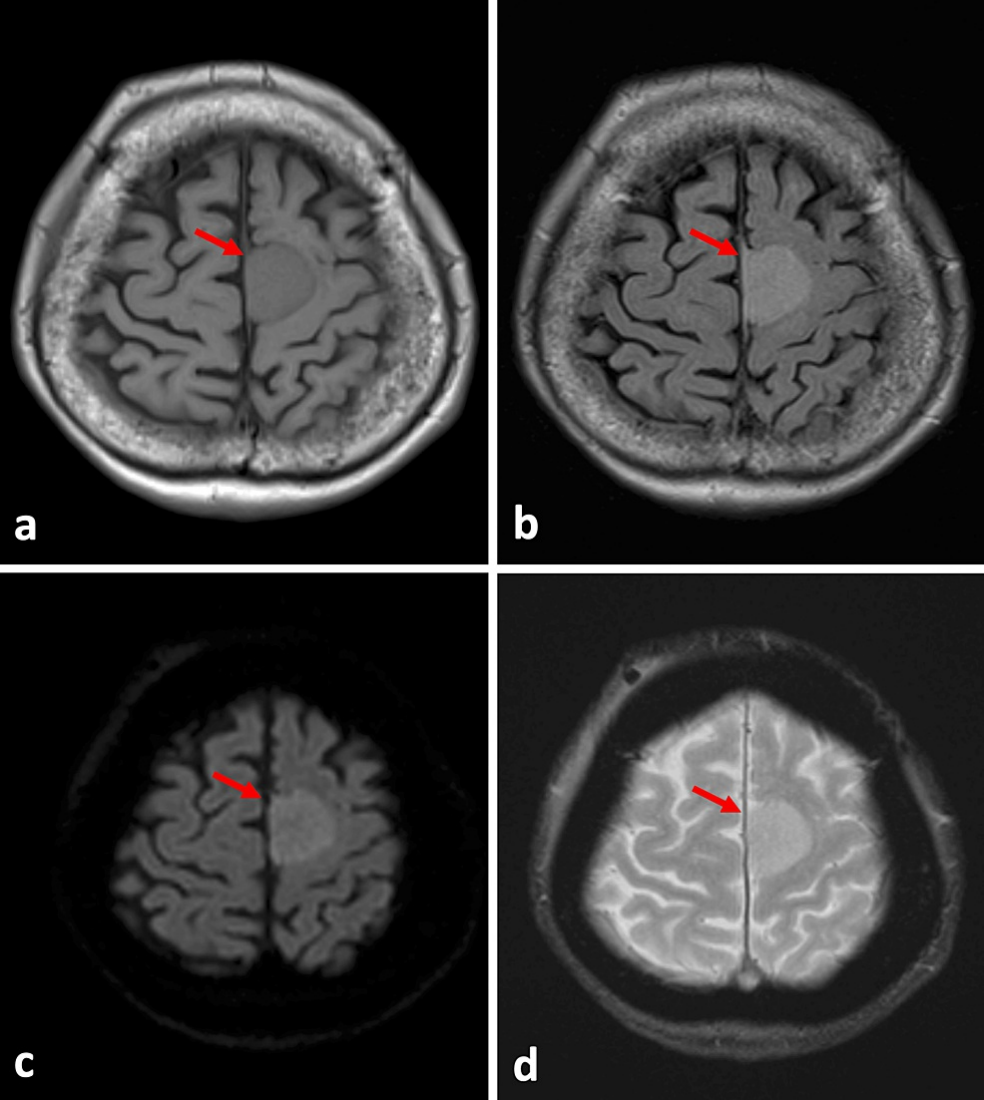
Results of magnetic resonance imaging one year before admission a: T1 weighted image; b: FLAIR; c: diffusion-weighted image; d: T2-star weighted image The tumor is in contact with the left side of the cerebral falx (red arrows). The internal signal appears uniform, and there are no indications of hemorrhage. FLAIR: fluid-attenuated inversion recovery

One year prior to her emergency visit, a brain tumor was incidentally detected on a head computed tomographic (CT) scan performed after a head contusion. Magnetic resonance imaging (MRI) performed at that time revealed a mass lesion attached to the cerebral falx with T1 iso-signal and hyper-signal on fluid-attenuated inversion recovery (FLAIR), which led to the suspicion of a left cerebral falx meningioma (Figure 1).

**Figure 2:**
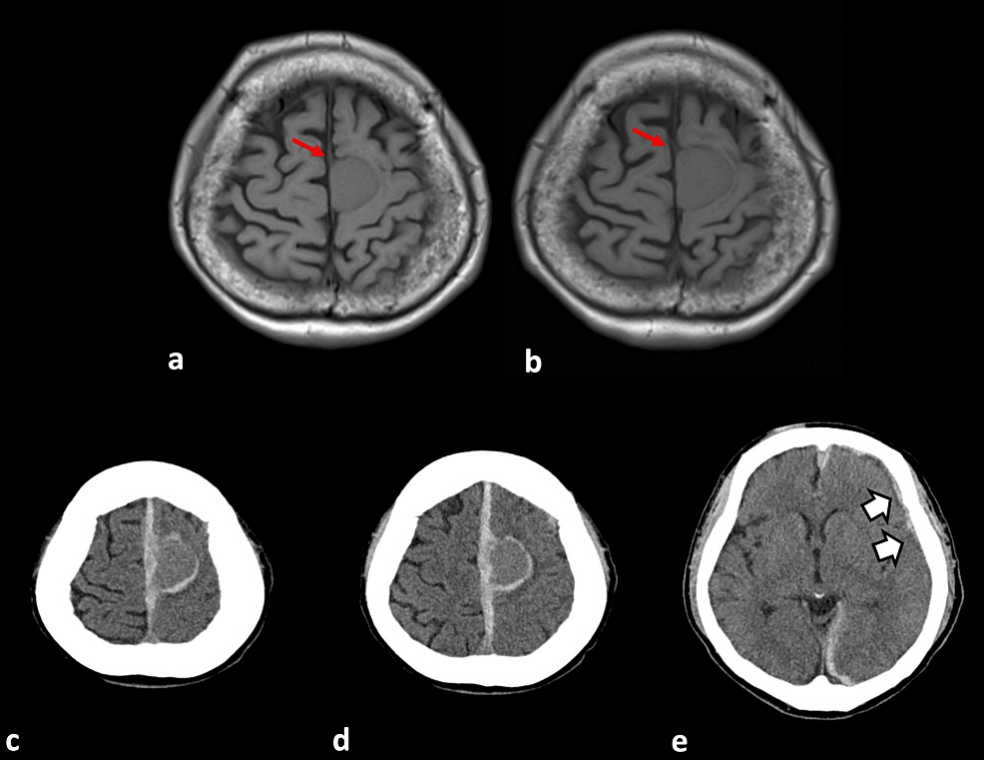
MRI T1-weighted images of follow-up (a,b) and CT images on admission (c-e) The MRI T1-weighted images depict the progression of the meningioma over time (red arrows), revealing an increase in tumor volume from 5.4 cm^3^ to 7.5 cm^3^. a: one year before admission; b: three months before admission; c-e: CT images on admission. There is an acute subdural hematoma along the cerebral falx and a hemorrhage around the tumor. A portion of the acute subdural hematoma extends into the convexity (white arrows).

**Figure 3:**
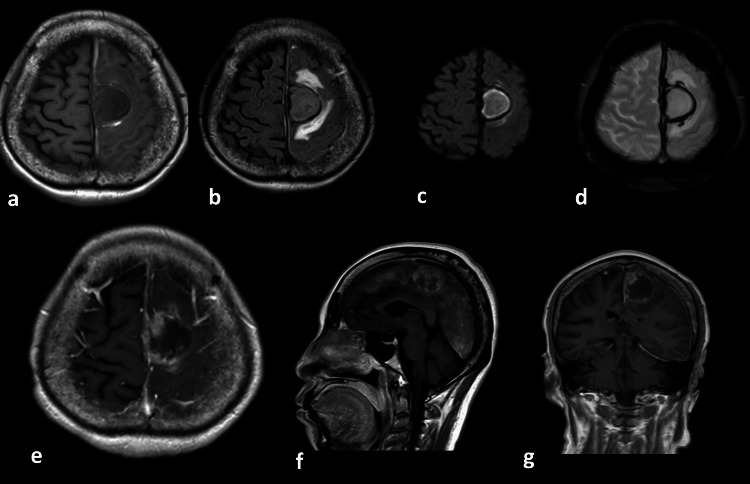
MRI images after hemorrhage a: T1 weighted image; b: FLAIR; c: DWI; d: T2-star weighted image; e-g: Gadolinium-enhanced MRI (e: axial image, f: sagittal image, g: coronal image) The images show brain parenchymal edema and hemorrhage around the tumor (b). A previously absent high signal on DWI was visible within the tumor (c). Enhancement was observed along the cerebral falx but not in most of the tumor's interior (e-g). FLAIR: fluid-attenuated inversion recovery; DWI: diffusion-weighted image

The tumor volume at that time was 5.4 cm^3^. A subsequent MRI scan conducted three months before the emergency visit revealed an increase in tumor volume to 7.5 cm^3^. Despite the increase in size, the patient remained asymptomatic and declined surgery. Therefore, the plan was to continue follow-up imaging. (Figure 2ab). The patient presented with mild paralysis of the right upper and lower limbs, which had persisted since the day before her emergency visit. On the morning of the presentation, she experienced a headache and increased paralysis of the right upper and lower limbs, prompting her to visit the emergency room. On arrival, her Glasgow Coma Scale score was 13 (E3V4M6), and right hemiplegia was observed. Computed tomography revealed interhemispheric acute subdural hematoma (ASDH), left hemispheric ASDH and surrounding tumoral hemorrhage (Figure 2cde). The CT angiography did not reveal any vascular malformations or aneurysms, and a diagnosed hemorrhage from a known tumor was revealed. Magnetic resonance imaging revealed a tumor with a T1 iso-signal to mild low signal with extensive surrounding edema. Gadolinium-enhanced MRI showed enhancement only in a portion of the tumor along the cerebral falx but not in the majority of the tumor (Figure 3).

Conservative treatment, including antihypertensive therapy, antiepileptic medication, and rehabilitation, was initiated because of the patient’s preserved level of consciousness and the mild mass effect of the hematoma.

The patient's paralysis was probably caused by the compression of the supplementary motor cortex by the tumor. Because paralysis could be improved by tumor resection, a decision was made to perform tumor resection with informed consent. On day nine of hospitalization, cerebral angiography revealed slight staining of the anterior superior portion of the tumor supplied by the left middle meningeal artery. The peripheral portion of this branch of the middle meningeal artery was embolized using 16.7% n-butyl-2-cyanoacrylate. On day 13, a tumor resection was performed. A left frontoparietal craniotomy was performed to access the interhemispheric fissure, and the space between the tumor and cerebral falx was dissected. The tumor was detached from the normal brain tissue and completely removed. The cerebral falx at the tumor attachment site was coagulated and cauterized, resulting in a Simpson Grade 2 resection. There was no obvious intratumoral hemorrhage, although there was a pre-existing hematoma and hemosiderin deposition around the tumor.

**Figure 4:**
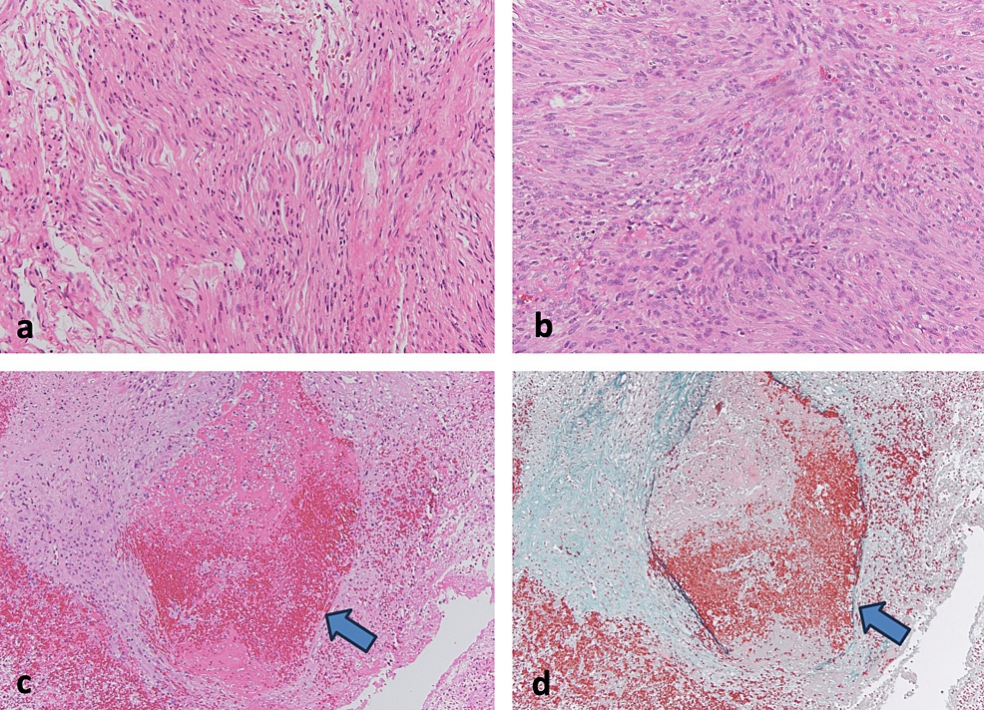
Pathological findings Hematoxylin and eosin staining (a, b) reveals a fascicular arrangement indicative of fibrous meningioma characterized by spindle-shaped cells, along with meningothelial meningioma demonstrating abundant cellular morphology. This coexistence leads to the diagnosis of transitional meningioma. Disrupted tumor vessels (blue arrows) are observed within the specimen (c) (hematoxylin and eosin staining), and the Elastica Masson stain shows fragile vessels lacking smooth muscle (d).

The pathological diagnosis was confirmed as a transitional meningioma (World Health Organization (WHO) Grade I, according to the 2016 Classification of Tumors of the Central Nervous System). The tumor contained necrotic tissue and numerous disrupted tumor vessels lacking smooth muscle. However, it is unclear whether preoperative embolization affected these findings, and the cause of the subdural hematoma remains undetermined (Figure 4).

Postoperative MRI confirmed complete tumor removal, and the edema of the brain parenchyma surrounding the tumor significantly improved. Right hemiplegia also improved over time, and the patient was able to walk two weeks after the surgery. On the 27th day after surgery, the patient was transferred to the rehabilitation hospital. The patient and her family provided consent for the publication of this case report.

## Discussion

Meningiomas are the most common type of primary brain tumor, accounting for 35.8% of all primary brain tumors [10]. Meningiomas are usually benign tumors that present with headaches, seizures, and focal neurological deficits. Hemorrhagic meningiomas are rare and occur in approximately 1.3% of all cases [11,12]. Leclerc et al. recently reviewed 120 patients with hemorrhage-onset meningiomas. Most cases (88%) were convexity meningiomas; 7% were posterior cranial fossa meningiomas; and 6% were intraventricular meningiomas. The most common type of hemorrhage was subdural hematoma (41%), followed by intraparenchymal (37%), subarachnoid (18%), and intraventricular (4%) hemorrhage. Pathologically, 79% of the cases were classified as WHO Grade I, with meningothelial meningioma being the most common (38%), followed by fibrous meningioma (19%). Patients with hemorrhagic meningiomas have a mortality rate of 20% and a morbidity rate of 21%. Among 19 patients who did not undergo meningioma removal during the follow-up period, 14 experienced rebleeding, with a median time to rebleeding of 120 days [1]. Studies comparing hemorrhagic meningiomas to all meningiomas have demonstrated that bleeding is associated with younger (<30 years) and older (>70 years) age, the presence of antiplatelet therapy, hypertension, and intraventricular or convexity localization [13]. Meningiomas can cause hemorrhage by several mechanisms, including direct vascular invasion by tumor cells [14,15], disruption of fragile vessels due to rapid angiogenesis and dilation of nutrient vessels [16,17], hemorrhagic changes due to extensive necrosis, and intratumorally infarction [17]. The disruption of subdural veins can lead to hemorrhagic changes [2]. Tumor removal is the recommended treatment for hemorrhagic meningiomas, with reports of rebleeding in four of five cases in which only hematoma removal was performed without tumor removal [1] and death in all 10 cases in which surgical resection was not performed [14].

Most meningiomas that develop from acute subdural hematomas are located in the convexity region.

Only nine cases of cerebral falx meningioma arising from acute subdural hematomas of the interhemispheric fissure have been reported, including the case presented here [2-9]. Table 1 summarizes the nine cases.

**Table 1:** Summary of nine cases of interhemispheric acute subdural hematoma associated with falx meningioma GR: good recovery; MD: moderate disability; SD: severe disability; ND: not determined

No.	Author	Year	Symptom	Treatment	Pathological diagnosis	Outcome	Causes of bleeding
1	Okuno [2]	1999	Vomit, hemiparesis, disturbed consciousness	Transarterial embolization, hematoma and tumor removal	Transitional meningioma	MD	Vessel rupture
2	Dallocio [6]	2003	Hemiparesis, disturbed consciousness	Tumor removal	Meningotheliai meningioma	MD	ND
3	Goyal [9]	2003	Headache	Hematoma and tumor removal	Transitional meningioma	GR	Vessel rupture
4	Krishnan [3]	2015	Headache, hemiparesis	Hematoma and tumor removal	Fibrous meningioma	GR	ND
5	Suzuki [7]	2018	Headache, hemiparesis	Transarterial embolization, tumor removal	Angiomatous meningioma	GR	Vessel rupture
6	Matsuoka [8]	2019	Headache	Tumor removal	Transitional meningioma	GR	Vessel rupture
7	Sato [4]	2020	Headache, hemiparesis	Transarterial embolization, tumor removal	Transitional meningioma	GR	Venous hypertension
8	Oyamada [5]	2022	Seizure, hemiparesis, disturbed consciousness	Hematoma and tumor removal	Meningothelial meningioma	SD	Vessel stretching
9	Present case	2023	Headache, hemiparesis	Transarterial embolization, tumor removal	Transitional meningioma	GR	Vessel rupture

Of the nine patients, six experienced headaches; seven experienced hemiplegia; and three experienced disturbances of consciousness. During the follow-up of previously identified tumors, hemorrhage occurred in two cases, including the present report. In the other seven cases, meningiomas were identified during close examination of acute subdural hematomas or intraoperatively. All patients underwent tumor resection, and four cases underwent combined preoperative embolization. Pathology results showed five cases of transitional meningioma, two cases of meningothelial meningioma, and one case each of angiomatous and fibrous meningioma. Of the nine patients, six had favorable outcomes.

A unique feature of this case was that the tumor volume had increased rapidly by more than 2 cm^3^ during the previous year. A previous report also described a case of hemorrhage during follow-up that demonstrated rapid meningioma enlargement [13]. Tumors with a clear tendency to enlarge may cause hemorrhage. Surgery should be considered more aggressively in this case because of the increased risk of hemorrhage and the appearance of local neurological symptoms. 

In the present case, the tumor was in contact with the supplementary motor cortex. The hemorrhage caused a rapid increase in pressure, resulting in rapid, progressive paralysis. Paralysis improved when the pressure was relieved by tumor removal, resulting in a good outcome. Determining the indications for the treatment of hemorrhagic meningiomas is challenging because of the severity of symptoms at onset. However, aggressive surgical interventions may prevent symptom exacerbations and improve patient outcomes.

## Conclusions

We present a rare case of falx meningioma that led to an acute subdural hematoma. Patients who experience apparent meningioma enlargement during follow-up may be at risk of hemorrhage, and aggressive surgical removal should be considered. Focal symptoms in patients with hemorrhage-onset meningiomas without intraparenchymal hemorrhage may be caused by pressure from tumors and hematomas. The possibility of postoperative recovery also favors more aggressive surgeries.
